# Investigating the Disparity in Visual Stimuli-Induced Behavioral Responses Between *Bactrocera dorsalis* and *Zeugodacus tau* (Diptera: Tephritidae)

**DOI:** 10.3390/insects17010008

**Published:** 2025-12-20

**Authors:** Fathelrahman Ahmed Naiem, Weiwei Zheng, Kamran Haider, Kamil Kabir, Imran Afzal, Hongyu Zhang

**Affiliations:** National Key Laboratory for Germplasm Innovation and Utilization for Fruit and Vegetable Horticultural Crops, Hubei Hongshan Laboratory, Institute of Urban and Horticultural Entomology, College of Plant Science and Technology, Huazhong Agricultural University, Wuhan 430070, China; naiemfath@gmail.com (F.A.N.); wwzheng@mail.hzau.edu.cn (W.Z.); kamranhaider345@gmail.com (K.H.); kamilkabir@webmail.hzau.edu.cn (K.K.); iafzal116@gmail.com (I.A.)

**Keywords:** behavior, *Bactrocera* species, LED light, attraction rate, sensitivity

## Abstract

Tephritid flies employ visual and chemical cues to locate and assess suitable habitats, food sources, mating sites, and ovipositional resources. *Bactrocera dorsalis* and *Zeugodacus tau* are economically significant pests that infest a wide range of fruits and fleshy vegetables. Understanding their visual sensitivity is crucial for developing effective and ecologically friendly management strategies. Thus, in this study, we compare the responses of these two species to various visual stimuli across laboratory, greenhouse, and closed-orchard environments. Our experiments reveal that, across the tested physiological states, both species exhibit a preference for certain wavelengths in the laboratory, particularly 520 nm and 560 nm. In the greenhouse, green and yellow models captured significantly more females of both species. *Z. tau* females showed greater sensitivity to the yellow model than *B. dorsalis* females. Additionally, *Z. tau* showed a higher affinity for both spherical and cylindrical shapes, while *B. dorsalis* flies were only attracted to the spherical model. In a closed orchard area, traps modified according to the best-performing combination used in the laboratory and greenhouse (shape and light) increased the capture counts of both species over time, with *Z. tau* exhibiting greater visual attraction sensitivity than *B. dorsalis*. These findings provide a theoretical and scientific foundation for improving trapping techniques targeting these two species.

## Simple Summary

In insects, the host-finding stage is crucial for identifying suitable oviposition sites, which are heavily influenced by visual cues. *Bactrocera*—true fruit flies—demonstrate a range of host preference habits across different species. We conducted a series of visual behavioral studies to investigate differences in sensitivity to key visual cues between two species, *Bactrocera dorsalis* and *Zeugodacus tau*, under controlled laboratory, greenhouse, and semi-field conditions. Our results show that both species exhibit high sensitivity to wavelengths of 520 nm and 560 nm, with higher responses to green and yellow light. Critically, *Z. tau* exhibited a stronger response to visual stimuli and further behavioral integration of multiple visual cues in the field. These findings underscore interspecific differences in visual cue processing that may contribute to ecological and visual behavioral divergence.

## 1. Introduction

Fruit flies of the family Tephritidae are globally recognized as highly destructive agricultural pests [1]. In these flies, the host-finding stage plays a crucial role in locating suitable oviposition sites and is strongly influenced by visual and olfactory cues [2,3]. Responses to these stimuli can either attract or repel the insects, depending on the nature and concentration of the stimulus [4]. Both vision and olfaction play important roles in host preferences, with vision having a greater effect than olfaction on short-range detection in some insects [5,6].

Compound eyes, serving as vital visual sensory organs, can discern colors and shapes [7], detect moving objects, and perceive the plane of polarized light [8]. Variations in the morphology of the compound eye affect the visual field and are likely to result in differences in behavior, lifestyle, and habitat preferences that impose distinct demands on the visual system [9]. At the molecular level, visual perception in Tephritid flies is mediated by opsin genes, which encode light-sensitive proteins located in the photoreceptor cells of compound eyes. These opsins determine the fly’s spectral sensitivity to ultraviolet (UV), blue, and green light, thus shaping its behavioral responses to visual stimuli [10,11,12].

The influence of visual cues on the attraction behavior of fruit flies has garnered significant attention in recent studies, which have revealed distinct responses to the color, shape, and size of presented stimuli [13,14,15]. Visual stimulants are already widely integrated into Tephritid IPM programs, where colored sticky cards and LED-based devices are designed to match trap spectra with insect visual sensitivities to enhance field capture efficiency [16]. Recent advances show that combining optimized visual cues such as narrow-band wavelengths, high-contrast surfaces, or programmable LED illumination with established lure-based traps can substantially increase detection sensitivity and overall trapping performance in operational field programs [17].

In field applications, traps equipped with visual attractants, such as yellow sticky panels, significantly improve Tephritid fly capture, underscoring the synergistic contribution of visual cues [18]. Generally, spherical shapes are reported to be more attractive to female western cherry fruit flies compared to cubes, cylinders, or rectangles [19]. López-Guillén [20] reported that the West Indian fruit fly displays stronger attraction to wavelengths of light ranging from 340 nm to 670 nm. Wang et al. [6] found that green is optimal for capturing *B. minax*. Additionally, Yee [21] observed that red spheres with a diameter of 10 cm attracted more *Rhagoletis indifferens* flies than red spheres with diameters of 8 or 12 cm and significantly more flies than yellow rectangles and spheres with diameters of 4 or 6 cm. Negative phototaxis has also been documented in other insect groups. For example, cocooned parasitoid wasp larvae strongly avoid short-wavelength light, demonstrating that wavelength-dependent aversion behaviors can occur in addition to positive phototaxis [22].

*Bactrocera dorsalis* Hendel (Oriental fruit fly) and *Zeugodacus tau* Walker (pumpkin fruit fly) are major invasive pests of significant agricultural and quarantine concern due to their polyphagous nature and high dispersal capacity [23,24]. Their host ranges, however, are markedly different. *B. dorsalis* is an extreme generalist, reported on over 300 hosts and recognized as a particularly damaging pest in China and other Asian regions [25,26]. In contrast, *Z. tau* has a narrow host range concentrated within the Cucurbitaceae, where it causes significant vegetable losses [24,27,28]. *B. dorsalis* has a smaller eye width and a smaller individual ommatidial area than *Z. tau*, but it possesses the greatest number of ommatrichia. In contrast, *Z. tau* has the lowest number of ommatrichia [9].

Numerous comparative studies on closely related Tephritid species have largely emphasized olfactory mechanisms, documenting species-specific differences in volatile preferences, antennal sensitivity, and odor-mediated host localization [29,30]. Although methyl eugenol and cue-lure remain central to male-targeted surveillance for *Bactrocera* and *Zeugodacus* species, their male specificity highlights the need for improved visual systems capable of attracting females as well [31]. This body of work has greatly advanced knowledge on fruit-fly chemical ecology, but fewer studies have examined how closely related species differ in visual sensitivity, even though vision is a primary modality of close-range host evaluation. The visual cues strongly influence female host-finding behavior; however, the development of effective female-targeted visual traps depends on understanding species-specific spectral preferences, making this gap particularly important. Despite the recognized importance of visual cues in Tephritid behavior, existing research rarely compares visual responsiveness across species or evaluates whether these differences remain consistent across varying ecological conditions.

To address this gap, this study was designed to systematically compare the visual responsiveness of the two species across three environmental conditions providing increasing ecological realism. Controlled laboratory assays were used to quantify baseline wavelength-dependent phototaxis, greenhouse experiments facilitated evaluation of color and shape preferences under semi-natural light conditions, and semi-field orchard trials tested whether the most attractive visual cues identified in previous tests remained effective under field-simulating conditions. Male flies were not included in this study, as the focus was on female responses to visual stimuli, which are crucial for host location and oviposition behavior. We identify the variations in wavelength and shape that affect both fly species’ use of spectral cues in host location and oviposition. This stepwise experimental framework provides a logically structured approach to determining whether these species’ differences in visual sensitivity are consistent across environments and whether these differences underlie their distinct host preferences.

## 2. Materials and Methods

### 2.1. Experimental Flies

The *B. dorsalis* and *Z. tau* individuals used in this study were acquired from laboratory-maintained cultures at Horticultural and Urban Entomology, Huazhong Agricultural University, PR, China, and reared in 30 × 30 × 30 cm screened cages. The flies were provided water (via cotton swabs) and artificial diets (sucrose/yeast powder, 3:1 ratio). Sexes were separated to ensure the females remained unmated (1–2 days old), and the flies were reared in sex-specific 30 × 30 × 30 cm cages until reproductive maturity. Mated females were obtained by housing males and females together for 48–72 h during the peak mating period. Mating was verified by direct observation of copulation, and only females observed in copula were used. The flies were maintained at 27 ± 1 °C under a 12 h light/12 h dark period [32].

### 2.2. Wavelength and Intensity

Based on prior studies on phototaxis in Tephritidae [28,33,34], we used LED lights with wavelengths of 454, 480, 520, 560, 580, and 620 nm. The wavelengths and irradiances of these LED lights were measured with a spectrometer (USB4H11915, Ocean Optics Co., Ltd., Florida, USA). All lights were sourced from Shenzhen New Photoelectric Technology Co., Ltd., Shenzhen, China. A digital lux meter (GM1020-CH-00, Shenzhen Jumaoyuan Science and Technology Co., Ltd., Shenzhen, China) was applied to determine the luminance intensity (lux) of the LED lights (Table 1).

### 2.3. Phototaxis Test Model

**Figure 1:**
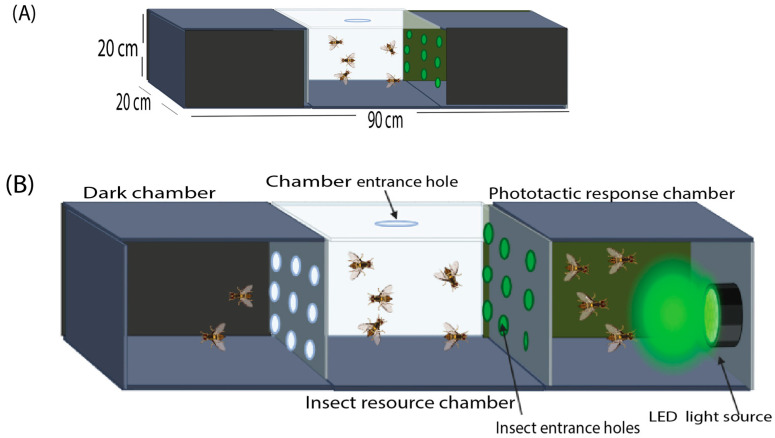
(**A**) Schematic representation of the phototactic response device, showing the overall dimensions (20 × 20 × 90 cm) and the arrangement of the three acrylic chambers. (**B**) Structural layout of the individual chambers (20 × 20 × 30 cm each), including the nine interconnecting entrance holes (2 cm diameter) and the positioning of the monochromatic LED light source within the phototactic response chamber.

The phototactic response device (Figure 1A,B) measured 20 × 20 × 90 cm. It was constructed from three connected transparent acrylic chambers, each with identical dimensions of 20 × 20 × 30 cm. These chambers were arranged sequentially to form (1) a dark chamber, (2) a central insect resource chamber, and (3) a phototactic response chamber.

Both the dark chamber and the phototactic response chamber were wrapped with opaque cork sheets to block ambient light and maintain accurate monochromatic light conditions during testing. In contrast, the central resource chamber was left uncovered to maintain a semi-natural ambient environment, allowing the flies to acclimate to natural light while remaining within a controlled experimental setup.

The chambers were separated by an opaque partition with nine circular entrance holes, each 2 cm in diameter. These holes enabled unrestricted movement of insects among the three compartments.

A monochromatic LED light source was mounted on the rear wall of the phototactic response chamber and connected to a rheostat to regulate and calibrate light intensity. During observations, the cork coverings on the top surfaces of the dark and phototactic response chambers were temporarily removed to allow direct visibility and accurate insect counting.

### 2.4. Responses of B. dorsalis and Zeugodacus tau to Varied Wavelengths in Laboratory Conditions

To determine the specific wavelengths preferred by the oriental and pumpkin fruit flies, their phototactic responses were estimated under set light wavelength and fly status conditions.

To compare the changes in light preference that occur after sexual maturation or mating in females of these species, immature (2 days old) and sexually mature (10 days old) females and mated and unmated females of the same age (14 days old) were selected as test subjects and exposed to different wavelengths of light.

The responses of the tested physiological states were evaluated separately in independent experiments, with each replicate comprising 30 flies. Each experiment was replicated 5 times. No fly was tested more than once. The control groups were tested in complete darkness.

Before each bioassay, we kept the tested flies in the dark for 2 h and then released them through the insect entrance hole of the resource chamber of the phototactic model.

The phototaxis rate of flies in each test was evaluated using the following formula:

Phototaxis rate (%) = (No. of flies in phototactic response chamber) × 100 (No. of flies in all chambers)

All laboratory tests were conducted at 17:00 in a well-lit room (200 lux), under controlled conditions of 27 °C temperature and 75% relative humidity. The behavior of the tested flies was observed 30 min after the light was switched on. After each trial, the testing apparatus was thoroughly cleaned with 75% alcohol and dried to eliminate any odors or residues left by the previous flies.

### 2.5. Response of Gravid Females of Two Species to Different Colors and Shapes in the Greenhouse

The responses of gravid females of the tested species to models of various colors were assessed using Styrofoam spheres with diameters of 10 cm. The spheres were wrapped in Tangle-Trap (Zhangzhou Enjoy Agricultural Technology Co., Ltd., Zhangzhou, China), a sticky adhesive applied to capture the flies upon contact that did not affect the visibility or coloration of the fruit models, ensuring that the flies’ responses were based solely on the visual properties of the colors. The color of the Styrofoam spheres was defined using a colorimeter camera to capture RGB values under natural sunlight. The use of RGB values for color data may have limitations compared to reflectance spectra measurements or controlled lighting conditions. The range of color models (500–600 nm) was chosen based on the results of previous laboratory experiments.

A total of twenty potted citrus trees were arranged inside a controlled greenhouse (5× 5 × 4 m) facility. The experimental layout consisted of five rows, each containing four colored models (one model per potted tree), with each model representing a distinct color treatment. The distance between potted trees within each row and between adjacent rows was maintained at 60 cm. Colored models were hung from the branches of the potted trees at 1.2 m above ground level to ensure consistent exposure across all replicates. Female flies of each species were examined separately. and 200 gravid females of each species per replicate were released inside the greenhouse. The number of flies captured by each colored sphere was recorded after 24 h. The experiment was conducted under controlled environmental conditions, with the temperature maintained at 27 ± 2 °C, relative humidity at 70 ± 3%, and natural light intensity varied between 400 and 1200 lux throughout the day. Before release, flies were acclimated inside mesh cages (40 × 40 × 40 cm) placed within the greenhouse conditions for one hour to allow them to adjust to the ambient temperature, light, and humidity conditions.

The response of the examined species to models of different shapes with equivalent surface areas was evaluated. The dimensions of the models were as follows: spherical models had a diameter of 10 cm, ovoid models measured 10 cm in length and 8 cm in width, cylindrical models had a height of 10 cm and a diameter of 6 cm, and pear-shaped models were 10 cm in height with a base diameter of 6 cm. All models were made from smooth Styrofoam, ensuring uniform surface properties across all shapes. The texture and gloss of the models were consistent to avoid any confounding effects on the flies’ behavioral responses.

The shape models were assigned to the potted trees following the same arrangement and environmental conditions described in the color test, using the same replication protocol and number of flies released per species for each replicate.

To avoid interference from residual flies remaining from the color-attraction experiment, the flies used in this test were marked on the thorax with a small spot of vinyl paint before the test. This mark did not interfere with fly behavior [35]. Gravid females of each species were tested separately, and 200 individuals were released into the greenhouse for each trial. The number of flies captured by each shape was recorded after 24 h of exposure.

### 2.6. The Response of Gravid Female Flies to the Most Attractive Visual Stimuli in a Closed Orchard

The semi-field orchard experiment was designed with two primary objectives. First, we aimed to determine which of the visual cues identified in the previous laboratory assays were most effective for attracting gravid *B. dorsalis* and *Z. tau* females under field-simulating conditions. Second, after identifying the most attractive cue combination, we aimed to use these optimal visual elements in a standardized trap to compare the visual sensitivity and behavioral responsiveness of the species across different time intervals in a semi-natural environment.

Semi-field experiments were conducted inside a closed, roof-netted citrus orchard enclosure measuring 20 × 5 × 4 m. The enclosure was fully covered with fine insect-proof mesh on the roof and all lateral sides, allowing natural airflow while preventing insect escape. Ambient lighting followed natural daylight cycles, and light intensity was recorded at three time points each day (05:00, 12:00, and 17:00) using a digital lux meter placed at trap height. Irradiance ranged from 780 to 920 lx at dawn, 1100–1300 lx at midday, and 450–600 lx in late afternoon, ensuring that all replicates experienced comparable light conditions. No external artificial light sources were present other than the LED units integrated into the experimental traps. The temperature ranged between 27 and 28 °C, with relative humidity between 67 and 70%.

Three modified electric insect-killer traps equipped with the most attractive visual cues from the laboratory tests were used to evaluate the flies’ responses under semi-field conditions. Four trap treatments were tested: (1) light only, (2) model + light, (3) model only, and (4) transparent panel trap (control). To avoid visual obstruction and ensure equal visibility among traps, we installed the traps 2 m above ground and spaced them at least 4 m apart. Each trap was placed 1.5–2 m away from the nearest citrus foliage, to ensure the traps remained clearly visible to the flies from multiple directions without interference from surrounding vegetation.

To ensure that airflow conditions did not bias trap visibility or insect movement, ambient wind conditions were monitored using local field weather forecasts one day before each trial and verified again immediately before the experiment. During the trials, airflow inside the enclosure was checked periodically, and any replicate in which unexpectedly high airflow occurred was canceled and rescheduled. Under acceptable conditions, airflow inside the netted orchard ranged between 0.2 and 0.5 m/s, ensuring that the air remained sufficiently mixed without creating directional currents that could influence fly or odor dispersal. For each replicate, 300 gravid females of each species were marked with a unique thoracic color code and released from a central point located approximately 5 m from all four traps. Release was performed at canopy height (1.5 m) to allow natural flight initiation. Distinct color markings were used for every replicate to ensure accurate individual identification, and the enclosure was checked after each trial to confirm that no residual insects remained.

At the start of each trial, the trap lights were switched on at 05:00 AM, and the flies were allowed to respond under natural semi-field conditions. After 24 h, all flies captured by each trap were counted to estimate the behavioral response. This procedure was repeated four times, with a minimum interval of 48 h between replicates to allow for environmental resetting and to ensure that no marked individuals from previous trials remained.

To directly compare species-specific sensitivity to visual cues, the most attractive cue combination identified from the preliminary trap test was installed into four standardized traps and arranged using the same spacing and environmental conditions. Another batch of 300 marked gravid females of each species was released at night under dark conditions. Their responses were recorded 20 min, 4 h, and 24 h after the trap lights were activated at 05:00 AM, facilitating assessment of both immediate and prolonged attraction patterns under semi-natural conditions.

### 2.7. Statistical Analysis

The experimental data were transformed with √ (x + 0.5) to stabilize variances among the treatments and normalize the data distribution. Laboratory data were analyzed using a three-way ANOVA. One-way ANOVA and Student’s *t*-test were used to compare the response to the color and shape of the models in the greenhouse between the species. The sensitivity of the two fly species to visual stimuli in a closed orchard was assessed using one-way ANOVA. Statistical analysis of all experimental data was conducted using SPSS Version 16.0 (SPSS Inc., Chicago, IL, USA), ensuring the reliability and accuracy of the results.

## 3. Results

### 3.1. Attraction Rate of Sexually Immature and Mature Females

**Figure 2:**
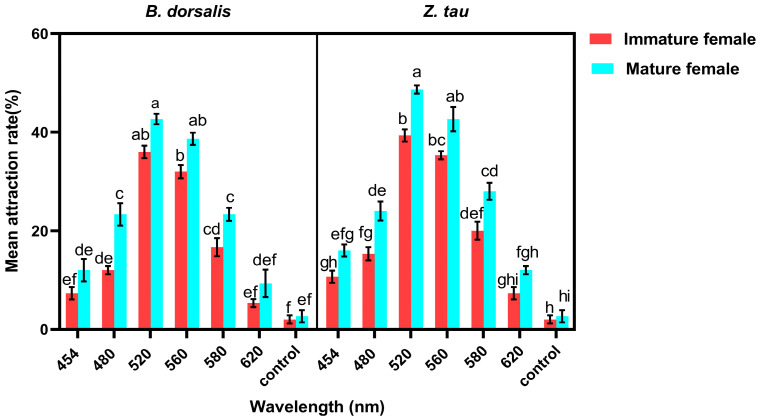
The mean attraction rate (%) of immature and mature *B. dorsalis* and *Z. tau* females across different wavelengths (454–620 nm) and a no-light control. Values represent mean ± SE (*n* = 5). Three-way ANOVA revealed significant effects of wavelength, species, and age status (*p* < 0.001). Different lowercase letters indicate significant differences among wavelengths within each species (Tukey’s HSD, *p* < 0.05).

The effects of species, sexual maturity, and wavelength on the phototactic response of *B. dorsalis* and *Z. tau* showed significant differences (species: F_1,112_ = 27.27, *p* < 0.001; state: F_1,112_ = 105.48, *p* < 0.0001; wavelength: F_6,112_ = 401.54, *p* < 0.0001). Mature females consistently showed higher responsiveness than immature females, and *Z. tau* displayed higher overall attraction than *B. dorsalis*. Across the spectral range, both species exhibited their strongest responses at 520 nm and 560 nm, with mean response rates for mature females reaching approximately (43 ± 2.0) and (37 ± 2.0) in *B. dorsalis* and (50 ± 2.0) and (45 ± 2) in *Z. tau*, respectively. The state × wavelength interaction was significant (F_6,112_ = 3.38, *p* = 0.0040), indicating that the greatest difference between mature and immature females was observed at peak wavelengths. The species × wavelength (F_6,112_ = 1.96, *p* = 0.0770), and species × state × wavelength interactions (F_6,112_ = 0.18, *p* = 0.983) were not significant, demonstrating that the two species shared a broadly similar wavelength–response profile (Figure 2).

### 3.2. Attraction Rate of Unmated and Mated Females

The effects of mated status and wavelength on the phototactic response of *B. dorsalis* and *Z. tau* revealed significant differences (species: F_1_,_112_ = 33.35, *p* < 0.0001; wavelength: F_6_,_112_ = 97.37, *p* < 0.0001). *Z. tau* exhibited higher overall attraction than *B. dorsalis*, and both species showed the strongest responses to 520 nm and 560 nm. At 520 nm, the mean attraction rate was 40 ± 2.1 for *B. dorsalis* and 46.66 ± 1.8 for *Z. tau*. At 560 nm, the mean attraction rates for *B. dorsalis* and *Z. tau* were 37 ± 2.1 and 45 ± 2.0, respectively. The attraction rates declined sharply at 454 nm and 620 nm, and control responses remained minimal.

**Figure 3:**
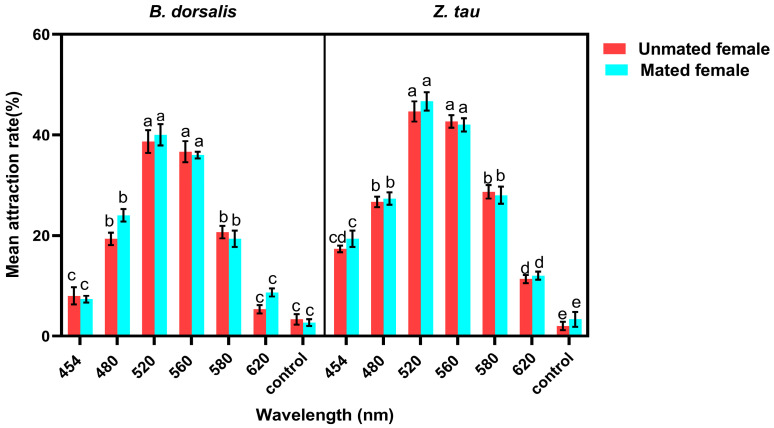
The mean attraction rate (%) of mated and unmated *B. dorsalis* and *Z. tau* across different wavelengths (454–620 nm) and a no-light control. Values represent mean ± SE (*n* = 5). Three-way ANOVA revealed significant effects of wavelength, species, and mating status (*p* < 0.001). Different lowercase letters indicate significant differences among wavelengths within each species (Tukey’s HSD, *p* < 0.05).

Mating state alone did not significantly affect attraction (F_1,112_ = 0.103, *p* = 0.7500), but a significant species × state interaction (F_1,112_ = 16.68, *p* < 0.001) indicated that the difference between mated and unmated females was slightly greater in *Z. tau* than in *B. dorsalis*. The species × wavelength interaction was also significant (F_6,112_ = 3.32, *p* = 0.0048), showing that the species differed mainly at the peak wavelengths, where *Z. tau* responded more strongly. No significant state × wavelength or three-way interactions were detected (F_6,112_ = 1.66, *p* = 0.1400), demonstrating that mating state did not alter the shape of the wavelength–response curve for either species (Figure 3).

### 3.3. Response of Mated Female Flies to Different Colors of Models

**Figure 4:**
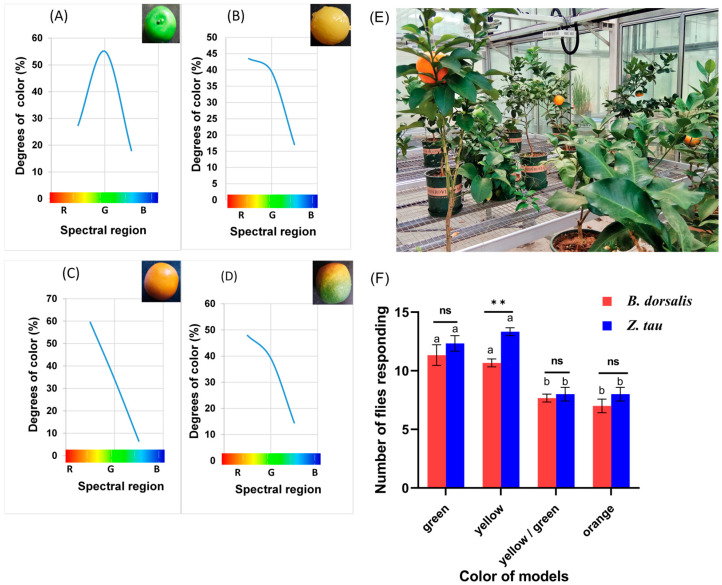
The colored models, (**A**) green, (**B**) yellow, (**C**) orange, and (**D**) yellow mixed with green, were photographed in sunlight using a colorimeter camera. The resulting JPEG images were analyzed for their RGB values. Each model was randomly hung on a branch of a potted tree in the greenhouse (**E**). Female flies were exposed to these different colored models, and their behavioral responses were recorded (**F**). Student’s *t*-test was used to compare the average response values (mean ± SE) between the two fly species, and the number of flies responding to color was statistically analyzed using one-way ANOVA. Bars sharing the same letter indicate no significant difference according to the Tukey HSD test (*p* < 0.05, ** *p* < 0.001; ns = not significant; *n* = 5).

The responses of female *B. dorsalis* (F = 22.9; df = 3, 8; *p* = 0.0003) and female *Z. tau* (F = 9.66; df 3, 8; *p* = 0.0049) were significantly influenced by the color of the tested models, as shown in (Figure 4). Green and yellow models elicited the highest capture rates in both species. For the green model, mean captures were (11.33 ± 0.88) for *B. dorsalis* and (12.33 ± 0.67) for *Z. tau*, while for the yellow model, captures were 10.67 ± 0.33 and 13.33 ± 0.33, respectively. These responses were significantly greater than those to the yellow–green mixture and orange models (*p* < 0.05). Notably, *Z. tau* females exhibited a significantly stronger attraction to the yellow model compared with *B. dorsalis* (t = –5.66; *p* = 0.0013).

### 3.4. Response of Mated Female Flies to Models of Different Shapes

**Figure 5:**
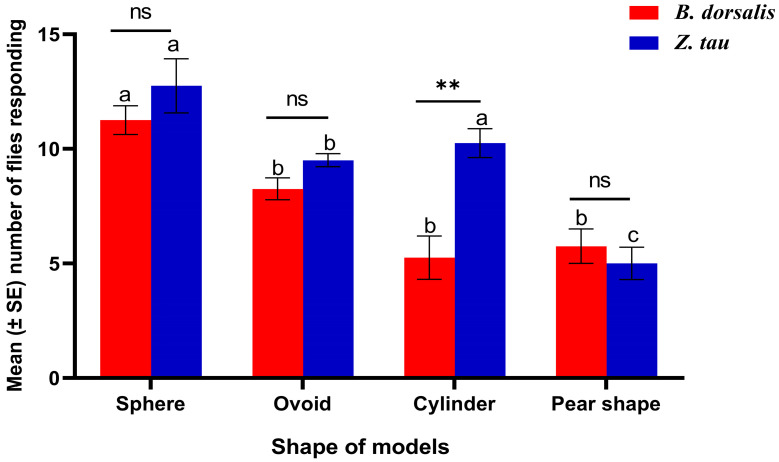
The number of flies (mean ± SE) responding to each model shape for both species. The responses of the two species were compared using Student’s *t*-test, while differences among model shapes were analyzed using one-way ANOVA for each species. Bars sharing the same letter indicate no significant difference according to Tukey’s HSD test (*p* < 0.05, ** *p* < 0.001; ns = not significant).

The shape of the tested models had a significant impact on the response of female *B. dorsalis* and *Z. tau* flies. Spherical models captured a significantly higher number of female *B. dorsalis* adults (11.25 ± 0.63) compared to ovoid, cylindrical, or pear-shaped models (F 11.2; df = 3, 12; *p* = 0.0009). However, no significant differences were observed among ovoid, cylindrical, and pear-shaped captures (*p* < 0.05). In the case of female *Z. tau*, both spherical and cylindrical models were more effective in capturing adults (12.75 ± 1.18) compared to ovoid and pear-shaped models (F = 17.7; df = 3, 12; *p* = 0.0001). Furthermore, the number of *Z. tau* individuals on cylindrical models was higher than the number of *B. dorsalis* individuals (t = 4.40; *p* = 0.0063) (Figure 5).

### 3.5. Response of Flies to the Most Attractive Light and Models in a Closed Orchard

**Figure 6:**
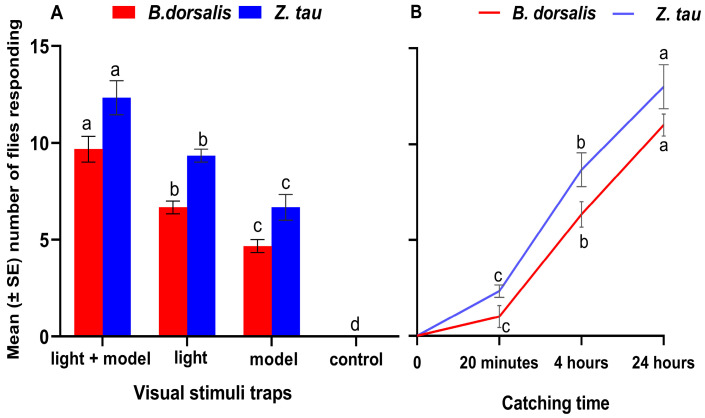
The number of flies (mean ± SE) responding to different traps (**A**), and the number of flies (mean ± SE) responding to visual stimuli per time in the natural arena (**B**). One-way ANOVA and bars sharing the same letter indicate no significant difference according to the Tukey HSD test (*p* < 0.05, *n* = 5).

The response of mated *B. dorsalis* and *Z. tau* to visual attractants in a closed orchard was examined (Figure 6A). The visual attractants tested had a significant impact on the response of both species (*B. dorsalis*, F = 227; df = 3, 8; *p* < 0.0001; *Z. tau*, F = 197; df = 3, 8; *p* < 0.0001). The traps equipped with both the green model and 520 nm light captured significantly more *B. dorsalis* (9.66 ± 0.66) and *Z. tau* (12.33 ± 0.88) individuals compared to traps with only light or traps with the green model and transparent panel trap (control) (*p* < 0.05).

The sensitivity (response per time) of mated *B. dorsalis* females (F = 227; df = 3, 8; *p* < 0.0001) and *Z. tau* (F = 197; df = 3, 8; *p* < 0.0001) to most visual attractants significantly increased with time (*p* < 0.05). However, the visual stimulus in the field captured more *Z. tau* flies compared to *B. dorsalis* flies at each time point (t = −4.5825, *p* = 0.0039; t = −4.3709, *p* = 0.0049; t = −5.9603, *p* = 0.0010) (Figure 6B).

## 4. Discussion

Most insects possess highly conserved visual pigments that exhibit spectral absorption sensitivity ranging from 350 to 700 nm, enabling them to perceive and respond to light within this range [36]. The phototactic response of insects varies among species and even within the same species, influenced by several abiotic or biological factors [37,38]. In our study, we observed that both *B. dorsalis* and *Z. tau* females exhibited a preference for specific wavelengths across all physiological states examined, with a notable affinity for 520 nm and 560 nm, which fall within the green region of the spectrum. These findings align with earlier research by Liu et al. [39], who noted that *B. dorsalis* adults displayed heightened attraction to green and yellow LED light, and research by Li et al. [28], who reported that wavelengths in the 515–604 nm range were optimal for trapping *Z. tau*.

In nature, the surface color of the leaves, flowers, and ripe fruits of host plants typically falls within the short-wavelength spectrum to which insects are considered sensitive, which might be the reason for tephritidae’s attraction to wavelengths in the green region [40,41,42].

The attraction rates of females of both species to specific wavelength ranges increased with their age. This finding corroborates previous findings in other insect species, such as *Helicoverpa armigera*, *Loxostege sticticalis*, and *Laodelphax striatellus*, which have shown increased sensitivity to visual stimuli with maturity [43,44,45]. The phototactic response of female *B. dorsalis* and *Z. tau* increased with age, likely because their compound eyes are not fully developed during the early imaginal stage, resulting in weaker sensitivity of the blocking pigment cells, particularly for short wavelengths in the blue and red spectra. Age may also influence phototactic tendencies due to variations in the level of locomotor activity among flies [46].

The results of our greenhouse study demonstrated that female *B. dorsalis* and *Z. tau* were attracted to the colored spheres tested. Furthermore, our data indicated that females of both fly species exhibited a strong response to green and yellow spheres. *B. minax* adults displayed greater attraction to spheres colored orange or green-yellow mixtures [47]. *A. obliqua* adults were shown to prefer spheres that were lime green, orange, and yellow [20]. Compared to brown, violet, black, blue, and white spheres, adult *A. ludens* showed a preference for yellow, green, red, and orange spheres. [48]. The observed preference of *B. dorsalis* and *Z. tau* for green and yellow colors might be linked to their fruit-seeking behavior, as many fruits exhibit green and yellow cues. Interestingly, *B. dorsalis* females exhibited greater attraction to spherical models, whereas *Z. tau* females showed a higher preference for both spherical and cylindrical shapes. Similarly, adults of *B. minax* and *A. obliqua* were more strongly attracted to spherical models [20,47]. Yee [15] observed that *R. indifferens* was captured more frequently by spherical models compared to cubes, cylinders, or rectangles. *Z. tau*’s attraction to the cylindrical model suggests a response to cues that potentially elicit food-seeking or host plant-seeking behavior. *B. cucurbitae* females, on the other hand, responded similarly to both hemispheres and hemicylinders [49]. The spherical form appears to be the most appealing for numerous Tephritid species, since it mirrors the host fruits that adults seek to find food, mating partners, and locations for oviposition [50]. The results of our closed orchard study demonstrated that the combination of visual stimuli (models and light) increased the attraction rate for both species, with the effect becoming more pronounced over time. Furthermore, *Z. tau* displayed greater sensitivity to these visual combinations than *B. dorsalis*. In arthropods, various aspects of the visual field, such as its dimensions, acuity, and sensitivity, can be influenced by factors like the size, shape, number of ommatidia, and surface texture of the compound eye [51].

Our results demonstrate that *B. dorsalis* and *Z. tau* exhibit distinct and consistent preferences for specific wavelengths and shapes. Incorporating these species-specific visual preferences into trap design can enhance the efficiency of monitoring tools in IPM programs. Optimized visual traps based on the specific wavelengths and shapes may improve detection accuracy, support more precise population monitoring, and contribute to targeted management strategies while reducing dependence on chemical controls.

## 5. Conclusions

In summary, *B. dorsalis* and *Z. tau* showed broadly similar wavelength preferences. Wavelengths of 520 nm and 560 nm proved highly effective in attracting females of both species. The green and yellow models were highly attractive to females of both species, while the *Z. tau* females exhibited significantly stronger attraction to the yellow model compared with *B. dorsalis.* Notably, the spherical models captured a significantly higher number of female *B. dorsalis* adults, and spherical and cylindrical shapes were more effective at attracting *Z. tau*. Overall, the pumpkin fruit fly *Z. tau* demonstrated a higher sensitivity to visual stimuli compared to *B. dorsalis*. These findings enhance our understanding of visual behavior in closely related species *B. dorsalis* and *Z. tau,* and may support future research aimed at developing environmentally safe and more effective strategies for monitoring and managing *B. dorsalis* and *Z. tau*.

## Figures and Tables

**Table 1:** Wavelength and irradiance of the LED lights under 200 lx.

Wavelength (mean ± SE)	454 ± 2.0	480 ± 2.0	520 ± 3.0	560 ± 2.0	580 ± 2.0	620 ± 3.0
Irradiance under 200 lx (mean ± SE) (μW/cm^2^)	29.6 ± 2.0	29.6 ± 3.0	29.8 ± 2.0	30.8 ± 3.0	31.2 ± 3.0	30.7± 2.0
Range of colors	450 nm	500 nm	550 nm	600 nm	650 nm	
	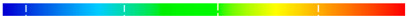					

## Data Availability

The data presented in this study are available on request from the corresponding author.
